# Defects in cell-cell junctions lead to neuroepithelial/ependymal denudation in the telencephalon of human hydrocephalic foetuses

**DOI:** 10.1186/1743-8454-7-S1-S56

**Published:** 2010-12-15

**Authors:** Montserrat Guerra, Deborah Sival, Antonio Jimenez, María Dolores Dominguez Pinos, Wilfred den Dunnen, Luis Federico Bátiz, José Manuel Pérez-Figares, Esteban Rodríguez

**Affiliations:** 1 Instituto de Anatomía, Histología y Patología, Facultad de Medicina, Universidad Austral de Chile, Valdivia, Chile; 2 Department. of Pediatrics, University Medical Center, University of Groningen, Groningen, The Netherlands; 3 Department. Biología Celular, Facultad de Ciencias, Universidad de Málaga, Málaga, Spain

## Background

In human foetal hydrocephalus and spina bifida aperta (SBA), the pathogenesis of abnormal cortical development is poorly understood. Previous reports have shown that neuroepithelial/ependymal denudation is involved in the neuropathogenesis of human foetal hydrocephalus and SBA. Interestingly, loss of the neuroepithelium/ependyma (denudation) at the Sylvian aqueduct is preceded by defective expression of adherent and gap junction proteins. In human foetal hydrocephalus, we aimed to investigate whether abnormal cortical development is similarly associated with intercellular ependymal defects at the telencephalic (sub)ventricular zones.

## Materials and methods

Human hydrocephalic foetuses were characterized according to their underlying pahogenesis [SBA (n=5, 21-40 weeks GA) and hydrocephalic foetuses with other congenital brain abnormalities (n=8, 12-40 weeks GA)] and studied by immunocytochemistry using antibodies against junction proteins (N-cadherin and connexin-43). Cilia (βIV-tubulin) and neuron/neuronal precursor (βIII-tubulin) markers were also used.

## Results and conclusions

In human hydrocephalic foetuses, we observed telencephalic subventricular zones with already denuded areas together with areas that were likely to undergo ependymal denudation (as shown by altered lining of neuroepithelial/ependymal cells). These areas were associated with abnormal expression of N-cadherin, formation of rosettes and periventricular heterotopias. In human foetal hydrocephalus, these findings support the concept that defective ependymal cell-cell junction proteins are related with abnormal neurogenesis and migration.

